# 

**Published:** 1893-06-10

**Authors:** 


### CONTENTS.

The International Hospital Congress at Chicago . . 161
Around the Hospitals . . . . . . . . . . . 162
Medical History from the Earliest Times. By E. T. WITHINGTON, M.A., M.B., Oxon. LVII.—The Clinicians . . 163
Contemporary Theory and Research . . . . . 164
Diet in Disease. By Mrs. ERNEST HART. XXII.—Underfeeding and Over-eating (*continued*) . . . . . . 165
Gleanings in Science and Medicine . . . . . . 166
Annotations.—A Reminder—"A Common Fault"—Ridiculous Fellows of the Royal Society . . . . . . . . . 167
Notes and News . . . . . . . . . . . . 168
The Hospital Clinic—
RHINOSCOPY. IV.—Examination of the Nose (*continued*) . . 169
BRISTOL GENERAL HOSPITAL.—Treatment of Club-foot . . 169
LONDON HOSPITAL.—The Methods and Applications of Skin Grafting . . . . . . . . . . . . 170
Editor's Letter Box.—The Uses of Hospitals . . . . 172
Festival Dinners and Meetings.—Paddington Green Children's Hospital . . . . . . . . . . 172
The London Hospital.—II. By OUR SPECIAL COMMISSIONER . 173
The Book World of Medicine and Science . . . . vii
Medical Vacancies and Appointments . . . . . x

### EXTRA SUPPLEMENT.—THE NURSING MIRROR.

News from the Nursing World.—The Royal Wedding—The Duchess of Connaught—The Nurse Dolls—Registered Nurses—The late Dr. Hadden—Without Impediments—Instruction for Mental Nurses—Appreciated Instruction—A Home at Bexhill—Cornwall—How to Secure Convalescence—Education in Dealing with Emergencies—Short Items—The Disabled Nurse . . . . . . . . . . ci

Sanitation for Nurses. By A SANITARY INSPECTOR. IX.—Some Recent Sanitary Provisions . . . . . . . ciii

Hospital Nursing in the North of India. By OUR INDIAN CORRESPONDENT.—II. In the Wards . . . . civ

More Work for Competent Women . . . . . . civ

Workhouse Infirmary Nursing Association . . . . cv

The Military Tournament, Islington.—The Hospital . cv

Ceylon.—The Civil Hospital, Colombo . . . . . cv

Wants and Workers . . . . . . . . . . cv

Everybody's Opinion.—Royal National Pension Fund for Nurses—Japan—Qualified Nurses—Manchester Infirmary—The Royal Wedding—Nurses as Patients—A Paying Cottage Hospital . . . . . . . . . . . . cvi

Appointments . . . . . . . . . . . . cvi

An Association of Nurses for the Insane . . . . cvii

Minor Appointments . . . . . . . . . . cvii

For Reading to the Sick.—Temptation . . . . . cvii

Notes and Queries . . . . . . . . . . . cvii

Sent.—II. Nearly Too Late . . . . . . . . cviii

Presentation . . . . . . . . . . . . . cviii

The Book World for Women and Nurses . . . . cix

